# Spatio-temporal gait variables predicted incident disability

**DOI:** 10.1186/s12984-020-0643-4

**Published:** 2020-01-30

**Authors:** Takehiko Doi, Sho Nakakubo, Kota Tsutsumimoto, Min-Ji Kim, Satoshi Kurita, Hideaki Ishii, Hiroyuki Shimada

**Affiliations:** Department of Preventive Gerontology, Center for Gerontology and Social Science, National Center for Geriatrics and Gerontology, 7-430, Morioka, Obu, Aichi 474-8511 Japan

**Keywords:** Frailty, Sarcopenia, Physical function, Mobility

## Abstract

**Background:**

Assessing the risk of disability in older adults is important for developing prevention and intervention strategies to decrease potential disability and dependency. The aim of this study was to examine the association between spatio-temporal gait variables and disability among older adults.

**Methods:**

We conducted a prospective study in a community setting. We collected data from 4121 subjects (≥ 65 years, mean age: 71.9 years). Gait speed, cadence, stride length, and stride length variability were measured at baseline. Participants were instructed to walk at their usual pace along a 6.4 m straight and flat path on which an electronic gait measuring device was mounted at mid 2.4 m. Subsequent disability was confirmed from long-term care insurance records.

**Results:**

During follow-up duration (mean: 49.6 months), 425 participants had incident disability. The cut-off value to detect high or low function in each gait variable was determined using the Youden index. Cox proportional hazard analysis adjusted for covariates showed that disability was significantly predicted by low function in each gait variable using the cut-off values: gait speed (hazard ratio [95% confidential intervals]: 2.06 [1.65–2.57]), stride length (2.17 [1.72–2.73]), cadence (1.49 [1.20–1.86], and stride length variability (1.46 [1.19–1.80]). The number of gait variables that scored in the low function category were also cumulatively related to subsequent disability (*p* < .001).

**Conclusions:**

This study revealed that spatio-temporal gait variables had a significant predictive value for incident disability. Multifaceted and quantitative gait analysis can contribute to disability risk assessment.

## Background

Disability is a condition that requires support to overcome difficulty or dependency in daily activities [1]. Diminishing disability duration and prolonging the duration of health with independence is essential for older adults [2]. An adequate risk assessment and preventive strategy are needed to accomplish this. Japan is becoming a super-aged society; therefore, methods for resolving age-related problems are needed [3].

Disability risk assessments for older adults are varied and multi-layered, with measures ranging from pathophysiological changes to behaviors along to geriatric syndromes. One useful assessment in a clinical setting is to evaluate physical function, especially lower-extremity function, which has good predictive value for subsequent disability [4]. Among physical functions, a robust gait is associated with decreased risk of adverse health events. Slow gait speed is a marker of disability [5, 6], hospitalization [5, 6], and mortality [7, 8]. Furthermore, slow gait is also an important factor in determining risk status such as frailty [9] and sarcopenia [10].

Evaluation of gait function using objective quantitative variables is recommended [11, 12]. Spatio-temporal gait variables assessed different constructs [13, 14] and had independent factors representing distinct gait domains based on factor analysis [15]. Traditionally, gait has been measured through such variables as speed; however, measuring gait variability has shown to have better discriminability regarding risk of failing [16] and frailty [17]. Furthermore, gait variability, which was independent from gait speed, predicted incident mobility disability, but not stride length and stance time [18]. However, it remains unclear which spatio-temporal gait variable is a good predictor of future disability in late life. Moreover, the cumulative effects of low gait function reflected in spatio-temporal parameters on disability risk are still unclear. The purpose of this study was to examine the relationship between quantitatively assessed spatio-temporal gait variables and subsequent disability incidence using a prospective cohort study.

## Methods

### Participants

Participants came from the population-based Obu Study of Health Promotion for the Elderly [19], which was a part of the National Center for Geriatrics and Gerontology – Study of Geriatric Syndromes [20]. The current study was designed as a prospective study. The baseline examination was conducted between 2011 and 2012. Inclusion criteria included: ≥65 years old when recruited for the study. A total of 15,974 individuals were found eligible for participation. Prior to recruitment, 1661 people were excluded because they had participated in other similar studies, were hospitalized or in residential care, or were certified at levels 3–5 to require support or care by the Long-Term Care Insurance (LTCI). A letter was sent to 14,313 individuals for the purpose of recruitment. A total of 5104 individuals participated in the baseline examination. The detailed recruiting protocol was previously described [20]. We excluded participants based on the following criteria: having any dependency for basic ADL, being certified at any level by LTCI, having specific medical conditions (stroke, Parkinson’s disease, Alzheimer’s disease), having severe cognitive impairment as assessed by a Mini-Mental State Examination (MMSE) score < 20, censoring due to moving away or death, and having missing values for any of these variables. Baseline assessments for medical conditions were conducted by well-trained nurses via face-to-face interview, and the other variables were assessed by well-trained staffs. Finally, 4121 participants were eligible for this study. During follow-up, we monitored incident disability using the records of LTCI.

### Spatio-temporal gait variables

The detailed protocol was described in previous studies [19, 21]. Participants were instructed to walk along a 6.4 m straight and flat pathway; five trials were conducted. Gait assessment was measured over 2.4 m, with 2 m allowed for acceleration and deceleration. An electronic measuring device (WalkWay MW− 1000, Anima Co., Tokyo, Japan) was mounted at the mid of pathway (2.4 m). The WalkWay is 800 mm wide, 2400 mm long, and 5 mm thick, and mounted with strain gages placed 10 mm apart (14,000 points). Gait speed was calculated from walking time measured by a stop watch, and the mean gait speed over five trials was expressed in meters per second. The other variables were cadence, stride length, and stride length variability acquired from the electronic measuring device. Based on a previous study that used a similar measuring device, spatio-temporal gait variables could be classified into three statistically independent factors representing distinct gait domains; the study also suggested the use of gait variables identified from the three factors [15]. Following the study, we selected variables from the three factors. Cadence was calculated by numbers of step per minutes and stride length was calculated by mean stride length (m) through trials. Variability was calculated by the coefficient of variation (CV) of stride length: CV [%] = [standard deviation / mean] × 100. When fewer than five strides of data points were acquired over five trials, the data were regarded as missing values.

### Disability

We monitored disability defined as LTCI certification for all subjects during the follow-up period (mean duration: 49.6 months). The LTCI was introduced in Japan and certifies a person as “Support Level 1 or 2” if needing support for daily activities or “Care Level 1, 2, 3, 4, or 5” if needing continuous care [22]. When certified by the LTCI, persons are eligible for various services, according to their nursing care plan, up to the maximum coverage amount for a municipality. A primary care medical doctor evaluates the need for LTCI and a trained healthcare official evaluates the need for nursing care, using a questionnaire that assesses current physical and mental status resulting from their medical conditions (e.g., treatments received or medication regimes). Subcategories of these items were paralysis and limitation of joint movement, movement and balance, complex movement, conditions requiring special assistance, conditions requiring assistance with activities of daily living/instrumental activities of daily living, communication and cognition, behavioral problems, and use of medical procedures during the previous 14 days. Based on the results, a computer program is used to calculate the applicant’s standardized scores for the seven dimensions of physical and mental status and estimated level of care and assign a care-need level based on the total estimated care-minutes. The Nursing Care Needs Certification Board (physicians, nurses, and other experts in health and social services appointed by a mayor) reviews whether the initial assessment is appropriate for each assessed individual, considering the applicant’s primary care physician’s statements and notes written by the assessor during the home visit. In this study, the outcome of disability was defined as a new certification of LTCI at any level during the follow-up period.

### Covariates

Covariates were selected as confounding factors based on selections in previous studies. A face-to-face interview was conducted by well-trained nurses to obtain participants’ information, including age, sex, height, weight, and presence of medical conditions (heart disease, diabetes mellitus, osteoarthrosis, and knee osteoarthritis). In addition, physical inactivity [19] and MMSE [23] were measured by well-trained staff. Physical inactivity was measured through responses to the following two questions: “Do you engage in moderate levels of physical exercise or sports aimed at health?” and “Do you engage in low levels of physical exercise aimed at health?” When participants answered “no” to both of these questions, they were evaluated as physically inactive.

### Statistical analysis

To examine characteristics associated with disability incidence, student-*t*-tests or χ^2^ tests were used to compare participant characteristics between individuals with disability and those without. To compare the degree of differences in gait variables between groups, effect size (Cohen’s d) was calculated: mean differences/pooled SD. Cohen d was suggested that threshold of large effect was 0.8, medium effects was 0.5, low effects was 0.2. To examine the association between gait variables, we calculated Pearson’s correlation coefficient among gait variables. Cox proportional hazards regression analysis was used to examine associations between each gait variable as a continuous variable and disability incidence. Analysis was conducted separately for each gait variable. Hazard ratios (HR) and 95% confidential intervals (CI) in each model were calculated. Disability was used as the objective variable and gait variables were separately used as explanatory variables, adjusted for covariates of age, sex, height, weight, medical condition, physical inactivity, and MMSE score. To examine the cumulative effects of low gait function reflected in spatio-temporal parameters, each gait variable was converted to categorical data (low function or high function) based on cut-off values. Cut-off values were calculated by using a receiver operating characteristic curve and the Youden index to determine sensitivity and specificity. Based on the cut-off values, participants were classified into either low function or high function categories for each gait variable. Then, the number of low functions for gait variables (gait speed, stride length, cadence, and stride length variability) were directly sored (0–4). For identifying associations of the number of low function with incident disability, the Cox proportional hazards regression analysis was used and adjusted model were also set in the same manner since gait variables were considered as continuous variables in the analysis. All calculations were performed using IBM SPSS Statistics, ver. 20 (IBM Corp., Armonk, NY, USA). Significance was defined as *P* < .05 for all analyses.

## Results

Comparison of participants with and without disability incidence are summarized in Table 1. All variables including spatio-temporal gait variables were significantly different between groups (all *P* < .01). Differences of gait variables between groups were medium to large based on effect size (*d*: gait speed 0.83; stride length 1.07; cadence 0.33; stride length variability 0.55). Correlation coefficients among gait variables are shown in the Additional file (Additional file 1: Table S1). All gait variables had significant correlations with the other gait variables (*P* < .001). Gait speed had a moderate-to-high correlation coefficient with stride length (*r* = 0.846) and cadence (*r* = 0.620), and stride length variability had a low-to-moderate correlation with with gait speed (*r* = − 0.366), stride length (*r* = − 0.389), and cadence (*r* = − 0.140).

**Table 1:** Comparison of characteristics between participants with and without incident disability

Variables	Without incident disability (*n* = 3696)		With incident disability (*n* = 425)		*P*
Age, years	71.2	(4.9)	77.6	(6.1)	< .001
Sex (women), %	52.6		60.9		.001
Height, m	1.56	(0.09)	1.52	(0.09)	.001
Weight, kg	57.3	(10.0)	54.5	(10.4)	< .001
Medical condition, %					
Heart disease	15.3		23.3		< .001
Diabetes mellitus	12.3		18.1		< .001
Osteoarthrosis	9.8		19.6		< .001
Knee osteoarthritis	13.7		18.1		< .001
Physical inactivity, %	28.0		36.7		< .001
Mini-Mental State Examination, score	26.5 (2.4)		25.5 (2.7)		< .001
Gait variables					
Gait speed, m/s	1.28	(0.19)	1.09	(0.24)	< .001
Stride length, m	1.26	(0.15)	1.10	(0.19)	< .001
Cadence, steps/min	124.7	(10.5)	121.2	(12.0)	< .001
Stride length variability, %	2.94	(1.50)	3.79	(1.87)	< .001

Values are presented as mean (SD) or proportion

Cox proportional hazards regression analysis showed that each gait variable was related with disability incidence adjusted for the covariates (HR [95% CI]: gait speed [per 0.1 m/s] 0.83 [0.79–0.87], *P* < .001; stride length [per 0.1 m] 0.77 [0.72–0.82], *P* < .001; cadence [per 1steps/min] 0.99 [0.98–0.99], *P* = .002; stride length variability [per 1%] 1.10 [1.04–1.16], *P* < .001). Analysis of the receiver operating characteristic curves showed cut-off values for each gait variable: (1) the cut-off value of gait speed was 1.10 m/s (AUC: 0.74 [95%CI 0.71–0.76]; sensitivity: 0.49; specificity: 0.84; *P* < .001); (2) stride length was 1.15 m (AUC: 0.75 [95%CI 0.72–0.78]; sensitivity: 0.41; specificity: 0.79; *P* < .001); (3) cadence was 116.5 steps/min (AUC: 0.58 [95%CI 0.55–0.61]; sensitivity: 0.66; specificity: 0.79; *P* < .001); and (4) stride length variability was 2.86% (AUC: 0.64 [95%CI 0.61–0.67]; sensitivity: 0.65; specificity: 0.56; *P* < .001). Based on these cut-off values, participants were classified into low function and high function categories for each gait variable. Participants were scaled 0 to 4 based on numbers of gait variables regarded as low function. For example, a subject (gait speed 1.0 m/s, stride length 1.0 m, cadence 110 steps/min, stride length variability 2%) had “3” gait variables regarded as low function (gait speed, stride length, cadence) and scaled “3”.

**Fig. 1:**
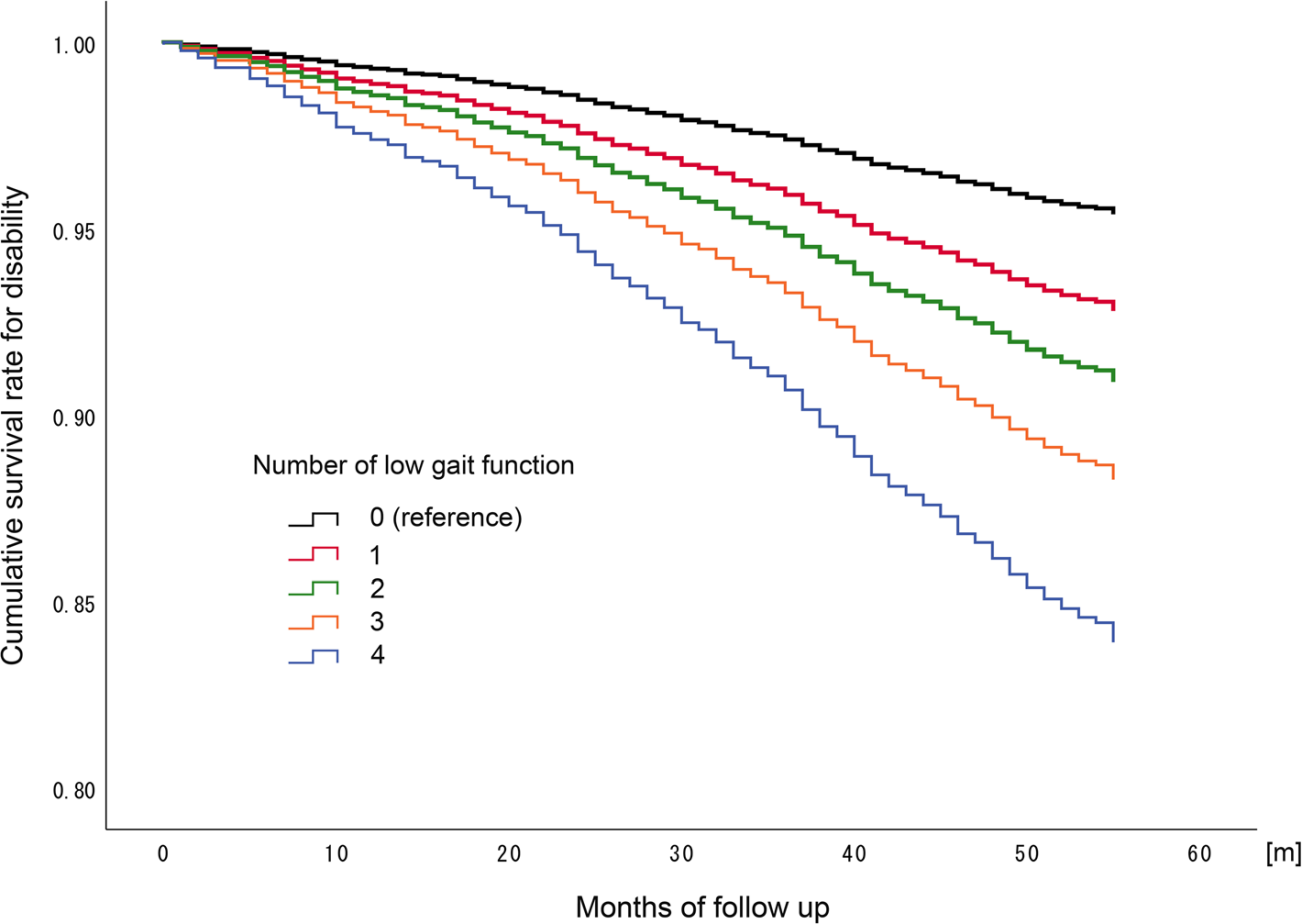
Cumulative survival rate for disability by number of measures assessed as low gait ability. Cox proportional regression analysis using number gait measures with lower gait ability based on respective cut-off values, adjusted for covariates: age, sex, height, weight, medical condition (diseases), physical inactivity and cognitive function

Results of the Cox proportional hazards regression analysis of the categorical data are summarized in Table 2. All gait variables were associated with disability, with low function of each gait variable predicting the risk of disability (all *P* < .001). The number of gait measures categorized as lower gait function was also associated with disability incidence, suggesting that the gait measures are a robust predictor of disability (Table 3 and Fig.1). When compared to a lack of low-function scores in the gait variables, participants with one or more gait variables categorized as low function showed a cumulative risk of disability (*P* < .01).

**Table 2:** Cox regression analysis of the relationship between gait variables and disability incidence

Gait variables	Cut-off value	Groups	Crude HR [95% CI]	*P*	Adjusted HR [95% CI]	*P*
Gait speed	1.10 m/s	Low function	4.66 [3.85–5.64]	< .001	2.06 [1.65–2.57]	< .001
		High function	Reference		Reference	
Stride length	1.15 m	Low function	4.86 [4.00–5.91]	< .001	2.17 [1.72–2.73]	< .001
		High function	Reference		Reference	
Cadence	116.5 steps/min	Low function	1.90 [1.55–2.32]	< .001	1.49 [1.20–1.86]	< .001
		High function	Reference		Reference	
Stride length variability	2.86%	Low function	2.26 [1.85–2.77]	< .001	1.46 [1.19–1.80]	< .001
		High function	Reference		Reference	

HR was calculated by Cox regression analysis for each gait variable based on individual cut-off values. The reference category is the high-functioning group from the analysis. Adjusted HR was calculated using covariates including age, sex, height, weight, medical condition (diseases), physical inactivity and cognitive function

*HR* hazard ratio, *CI* confidence interval

**Table 3:** The relationship between number of lower gait function measures and disability incidence

Number of lower gait function	Incident disability	Crude HR [95% CI]	*P*	Adjusted HR [95% CI]	*P*
4, *n* = 250	91, 36.4%	10.6 [7.66–14.6]	< .001	3.72 [2.58–5.35]	< .001
3, *n* = 379	92, 24.3%	6.47 [4.69–8.93]	< .001	2.64 [1.87–3.74]	< .001
2, *n* = 550	72, 13.1%	3.31 [2.36–4.65]	< .001	2.02 [1.43–2.86]	< .001
1, *n* = 1430	107, 7.5%	1.81 [1.33–2.48]	< .001	1.59 [1.16–2.17]	.004
0 (robust), *n* = 1512	63, 4.2%	Reference		Reference	

HR was calculated by Cox proportional regression analysis using number measures assessed as lower gait ability based on respective cut-off values. The reference category is 0 (number of lower gait functions) from the analysis. Adjusted HR was calculated using covariates including age, sex, height, weight, medical condition (diseases), physical inactivity and cognitive function

*HR* hazard ratio, *CI* confidence interval

## Discussion

This study revealed that spatio-temporal gait analysis can reliably predict subsequent disability. Quantitative measures of gait speed, cadence, stride length, and stride length variability were associated with disability incidence. Furthermore, the relationship was cumulative; higher numbers of gait variables rated as low function were associated with higher risk of subsequent disability.

Our results were consistent with previous studies. Although numerous studies focused on gait speed, in our study gait dysfunction was a good marker for health problems. Adverse health outcomes including disability [5] and mortality [7, 8] were predicted by slow gait speed. Disability among older adults is thought to be a consequence of frailty [24–26], which is regarded as a prodromal status of disability [9]. Slow gait speed is one of several markers for frailty [9, 27]. Our previous work showed that frailty or even pre-frailty in older people with slow walking speed increased the risk of future disability in community-dwelling older adults compared with individuals with a robust gait or those without frailty [28]. Similarly, slow gait has been used to define sarcopenia or severity of sarcopenia, which is a leading risk factor for disability [29, 30]. In fact, slow gait speed predicted incident disability [5, 6]. From a pooled analysis that included data from 27,220 community-dwelling older adults, gait speed was strongly associated with disability incidence, which is consistent with our results [31].

In our study, gait variables other than gait speed, i.e., stride length, cadence, and stride length variability, also had significant association with disability. The effect size of gait variables regarding differences between the groups were moderate to high. Gait speed and stride length had higher effect sizes with incident disability, while cadence and stride length variability had moderate effect sizes. The relationship between these gait variables and disability were less clear in previous studies. One earlier study showed that gait variability derived from stance time was independently associated with mobility disability, while other gait variables, including the mean and variability of step length, were not significant [18]. On the contrary, low trajectory of gait speed and step length were associated with incident disabling dementia [32], while gait variability and rhythm variables, including cadence but not pace variables including gait speed, predicted incident dementia [15]. The difference between the results of studies may be dependent on the difference between outcomes and different gait variables may have a linkage with different types of disability. However, disability in our data included both mobility and cognitive disabilities. To elucidate the linkage, further studies are required. Although a specific association between gait variables and cause of disability could not be identified, our results added to the evidence on the predictive value of spatio-temporal variables. Multifaced gait assessment using spatio-temporal gait variables could be useful for risk assessment of incident disability.

The cut-off values used to define the level of gait function in each of the gait variables were relatively high. Cumulative evidence showed various cut-off values of gait speed. Commonly used cut-offs for slow gait include 0.8 m/s [29] or 1.0 m/s [5] for risk assessment of disability, but some researchers suggest that each study should establish their own cut-off values based on the characteristics of their study cohort [33]. Accordingly, we calculated each cut-off from our participants’ gait measures. However, our sample study was conducted in a community setting and participants may have been younger than participants in other studies. Consequently, our cut-off values tended to be higher compared to other studies. Therefore, our cut points may be suitable for predicting future disability among community-dwelling individuals. Compared to the adjusted hazard ratios resulting from using cut-points from other studies, our study’s values were slightly higher (1.1 m/s [our study]: 2.06, 1.0 m/s: 1.89 [data were not shown], 0.8 m/s: 1.89 [data were not shown]). Other than gait speed, few studies have examined relevant cut-off values for cadence, stride length, or stride length variability. This knowledge would enhance our understanding of the clinical implications of gait measures, particularly in a community setting.

In addition, the cumulative effects of spatio-temporal gait measures for predicting disability risk were relevant. Gait measures acquired through gait analysis are varied and wide-ranging. Following the procedure used in a previous study [15], spatio-temporal gait variables were classified into pace factor, rhythm factor, and variability factor, based on factor analysis; in the previous study, gait speed and stride length were classified into pace factor, cadence into rhythm factor, and stride length variability into variability factor. Verghese et al. showed the different clinical implications of gait variables based on factors and usefulness of the single gait variables that were identified from the three factors [15]. Most of previous gait analysis studies using electronic measurements had relatively small samples; thus, the value of using gait measures to predict adverse health outcomes was still unclear. Using a large sample and longer follow-up duration, our study expanded knowledge on the clinical relevance of spatio-temporal gait assessment.

This study used a prospective design that disability incidence was captured monthly by monitoring disability diagnoses recorded in the LTCI system administered by the Japanese government. This procedure used a systematic methodology for evaluating individual status [34]. Thus, we could reliably determine disability incidence from the LTCI data. In addition, our study determined cut-off values in spatio-temporal gait variables and the cumulative effects of spatio-temporal gait measures for predicting disability risk. These findings have utility in clinical settings and are a strength of the study. Our study had also limitations. Disability in our data could not identify specific causes of disability, e.g., mobility disability or cognitive disability, or distinguish causes, such as dependence on upper limbs or lower limbs. Finally, gait analysis methodology using electronic devices is varied [11, 12]. Thus, different spatio-temporal gait variables from our study may also be useful for risk assessment. More studies are needed to identify which gait variables or gait analysis methods are most suitable for disability risk assessment. Further study is also required to clarify underlying pathophysiological pathways between gait and disability. Finally, our study was conducted among community-dwelling older adults with independency. Although participants had various medical conditions, cut-off threshold of gait variables was calculated based on community-dwelling older adults with independency. Further studies were required considering specific trend based on medical conditions in clinical settings.

## Conclusions

Spatio-temporal gait measures were significantly related to subsequent disability incidence during follow-up. The relationship was cumulative; higher number of gait variables that scored in the low function category were associated with increased risk of disability. Quantitative gait analysis to evaluate multifaceted gait ability contributes to our understanding of disability risk.

## Supplementary information

**Additional file 1: Table S1.** Correlation coefficients among gait variables.

## Data Availability

Data sharing is not applicable to this article.

## Abbreviations

CI: Confidential intervals
CV: Coefficient of variation
HR: Hazard ratios
LTCI: Long-Term Care Insurance
MMSE: Mini-Mental State Examination
