# Supplementary material for: Spatio-temporal gait variables predicted incident disability
Source: J Neuroeng Rehabil. 2020 Jan 30;17:11. doi: 10.1186/s12984-020-0643-4 (PMC6993362; doi:10.1186/s12984-020-0643-4)
Supplement: Supplementary file 1 — Additional file 1: Table S1. Correlation coefficients among gait variables. [file 12984_2020_643_MOESM1_ESM.pdf]

Table S1. Correlation coefficients among gait variables.

		Gait speed	Stride length	Cadence	Stride length variability
Gait speed	*r*	-	-	-	-
	P	-	-	-	-
Stride length	*r*	0.846	-	-	-
	P	< .001	-	-	-
Cadence	*r*	0.620	0.150	-	-
	P	< .001	< .001	-	-
Stride length variability	*r*	-0.366	-0.389	-0.140	-
	P	< .001	< .001	< .001	-

Values were Pearson's correlation coefficients and p values.
